# Physical and Chemical Properties, Biosafety Evaluation, and Effects of Nano Natural Deer Bone Meal on Bone Marrow Mesenchymal Stem Cells

**DOI:** 10.3389/fbioe.2022.891765

**Published:** 2022-07-15

**Authors:** Yongbo Li, Zhe Tan, Jixiang Zhang, Junhan Mu, Han Wu

**Affiliations:** Department of Orthopedics, China-Japan Union Hospital of Jilin University, Changchun, China

**Keywords:** bone marrow mesenchymal stem cells, nanoparticles, bone powders, osteogenic differentiation, bone tissue engineering

## Abstract

At present, bone-based products are abundant, and the main sources are bovine bone and pig bone, but there are few studies on the development of deer bone as a bone repair material. Deer bone has important osteogenic effects in the theory of traditional Chinese medicine. It is rich in protein, ossein, and a variety of trace elements, with the effect of strengthening tendons and bones. Nanomaterials and their application in the repair of bone defects have become a research hotspot in bone tissue engineering. In this study, nano-deer bone meal (nBM), nano-calcined deer bone meal, and nano-demineralized bone matrix were successfully prepared. It was found that the Ca/P ratio in deer bone was significantly higher than that in cow bone and human bone tissue, and deer bone contained beneficial trace elements, such as potassium, iron, selenium, and zinc, which were not found in cow bone. The three kinds of deer bone powders prepared in this study had good biocompatibility and met the implantation standards of medical biomaterials. Cell function studies showed that compared with other bone powders, due to the presence of organic active ingredients and inorganic calcium and phosphate salts, nBM had excellent performance in the proliferation, adhesion, migration, and differentiation of bone marrow mesenchymal stem cells. These findings indicate that nBM can be used as a potential osteoinductive active nanomaterial to enhance bone tissue engineering scaffolds with certain application prospects.

## 1 Introduction

Bone defects caused by trauma, inflammatory diseases, and tumors are in urgent need of repair (Wang and Yeung, 2017; Baldwin et al., 2019; Simpson et al., 2020). Untreated bone defects may be infiltrated by connective tissue (Li et al., 2019a). Therefore, these problems not only pose challenges to clinicians but also increase the need for bone reconstruction (Zhao et al., 2017; Ho-Shui-Ling et al., 2018). Various surgical approaches have been used to treat bone defects, such as autologous/allogeneic bone grafting, guided bone regeneration, and distraction osteogenesis (Khojasteh et al., 2013). However, several disadvantages limit the use of autologous bone grafting, including additional surgical procedures, insufficient supply, and donor-site morbidity. Despite these complications, it remains the gold standard for bone grafting (Tang et al., 2016; Przekora, 2019). Given the limitations of autologous bone grafting, allografts can overcome the above drawbacks, but it carries risks of infection transmission, immune response, and rapid resorption (Long et al., 2012). A xenograft is another possible approach because it is similar to human bone in morphological properties (Long et al., 2012). Xenografts can be obtained from different species, including cattle, pigs, camels, and ostriches. Among them, bovine bone is still the first choice for bone reconstruction (Ghanaati et al., 2014; Eliaz and Metoki, 2017; Sonmez et al., 2017). Although xenografts are widely sourced and simple to operate, they have low osteoinductive properties and have potential infectious disease problems, such as bovine spongiform encephalopathy.

Nowadays, abundant products are developed based on bone, and there are many commercial bone meal (BM) brands. These products are divided into two categories. The first category includes BM prepared by strong alkali treatment, high-temperature sintering annealing, and other processes. The main components are hydroxyapatite and other inorganic components with bone conductivity, such as Bio-Oss and Osteobiol. The former comes from cow bone and the latter from pig bone [bab 2065]. The second category is based on conventional Urist preparation methods, such as ether degreasing and hydrochloric acid decalcification. The main components are organic components of demineralized bone matrix (DBM), such as Grafton matrix and Xin Kangchen (XKC), both of which are derived from allogeneic bone. They contain a variety of osteogenic growth factors with osteoinductive properties, such as bone morphogenetic protein (BMP), insulin-like growth factor, and transforming growth factor (TGF), which can promote the migration and differentiation of bone marrow mesenchymal stem cells (BMSCs) and bone tissue regeneration (Urist, 1965; Urist and Strates, 2009). As is known, natural bone tissue is mainly composed of organic components (35%) and inorganic components (65%). The organic components mainly include collagen and cell growth factors, and the inorganic components are hydroxyapatite, various anions and cations (calcium, phosphorus, and magnesium), etc. However, few BM on the market contains both organic and inorganic components.

Deer bone, the skeleton of the deer family, as traditional Chinese medicine occupies an important position in China’s medical field. According to the Dictionary of Traditional Chinese Medicine, its pharmacological function is to strengthen the body and tendons and to treat limbs, arthritis, and muscle pain caused by rheumatism. Studies have shown that the long-term administration of deer BM can relieve joint pain in elderly patients and reduce the incidence of osteoporosis. Nowadays, with the development of biochemistry, pharmacology, cell biology, and other disciplines, the analysis of deer bone components has gradually improved, and deer bones have been increasingly widely used in scientific research and clinical work. Deer bone contains a large number of proteins, collagen, phospholipids, chondroitin, and phosphoprotein that are conducive to osteogenesis, promoting the synthesis of collagen and elastin, delaying bone absorption, and promoting endochondral osteogenesis *in vivo* (He, 2011; Ren et al., 2019). Guo et al. studied the nutritional components of sika deer BM and its therapeutic effect on osteoporosis in ovariectomized rats and measured the contents of amino acids, major and trace elements, and fatty acids in sika deer BM. The results showed that sika deer BM has a good effect for the treatment of osteoporosis (Guo, 2017). Deer bone also contains a large amount of minerals, such as calcium, magnesium, iron, zinc, potassium, copper, phosphorus, selenium, and other elements, covering almost all the nutrients required for the formation of human bone marrow. These provide a suitable microenvironment for bone regeneration, which helps to slow down the aging of the bone marrow, maintain the hardness and toughness of the bones, prevent osteoporosis, and reduce the risk of fractures (Lee et al., 2014; Quan et al., 2015). Wang et al. studied the effects of deer BM on bone microstructure and bone minerals in rats with osteoporosis after ovariectomy, and the results showed that deer BM improved the changes in bone microstructure and serological indexes caused by ovarian removal, suggesting that it can prevent and treat osteoporosis caused by decreased estrogen (Quan et al., 2015). Epimedium bone with deer BM as the main raw material has been reported to improve osteoporosis, and its effect on increasing bone trabecular structural components is very significant (Xudong, 2021). An et al. gave glucocorticoid-induced osteoporosis rats gavage of deer bone polypeptide, and the results showed that deer bone polypeptide can inhibit the imbalance of calcium and phosphorus metabolism induced by dexamethasone, reduce alkaline phosphatase (ALP), increase osteocalcin, inhibit bone resorption and promote bone formation, and improve the pathological changes and microstructure of bone in osteoporotic rats (Li-ping, 2016). In addition, several preclinical studies have shown that deer bone-related products, such as deer BM and deer melon polypeptide, can effectively relieve osteoporosis caused by ovariectomy in rats and promote osteoblast proliferation (He, 2011; Xue et al., 2021).

Due to its small particle size and large specific surface area, nanomaterials exhibit different properties from their monolithic state. Studies have shown that nanoscale materials can promote the proliferation, migration, and adhesion of BMSCs. Nanomaterials have unique microscopic biomimetic structures, good mechanical properties, and superior osteoinduction, osteoconduction, and other biological properties. Nanomaterials and their application in the repair of bone defects have become a research hotspot in bone tissue engineering in recent years (Laschke et al., 2010; Chae et al., 2013; Touri et al., 2013; Aryaei et al., 2014).

At present, there is no basic research on the development of deer bone as a bone repair material. It is innovative and promising to develop deer bone repair materials with nano-technology.

In this study, three kinds of nano-deer BM (nano-deer BM (nBM), nano-calcined deer BM (nCBM), and nano-DBM (nDBM)) were prepared and compared with two kinds of commercial BM (Bio-Oss, XKC) to analyze the differences in particle size, substance structure, and nutrient content among different BM. The biological safety of nano-sized deer BM was tested. At the same time, the effects of different BM on BMSC proliferation, adhesion, spread, migration, and differentiation were studied to evaluate the possibility of deer BM as a bone repair material in clinical application.

## 2 Materials and Methods

### 2.1 Materials

Bio-Oss was purchased from Geistlich Pharmaceuticals Ltd. (Switzerland). XKC was acquired from Beijing Xinkangchen Medical Technology Development Co., Ltd. (China).

### 2.2 Preparation of Nano-Deer Bone Meal

#### 2.2.1 Acquisition of Fresh Deer Bone

The fresh sika deer leg bones used in the experiments were fresh samples taken within 24 h after slaughter, which were provided by the breeding and slaughtering plant in Deer Town, Changchun City, Jilin Province. The soft tissue, fascia layer, periosteum, and marrow in the medullary cavity attached to the surface of the deer leg bone were removed layer by layer, and the articular cartilage around the joint was removed as much as possible, rinsed three times with sterile distilled water, and then cut using a grinding drill into bone blocks with a size of 2 cm×2 cm. The bone blocks were stored at −80°C for at least 6 h and then thawed in a 37°C water bath. This was repeated for three cycles. The above process was physical freeze-thaw decellularization. The bone blocks were then placed in 1% TritonX-100 and treated with a shaker at a constant speed (100 rpm) for 12 h. The name of this step was chemical decellularization. The decellularized bone blocks were thoroughly washed with sterile distilled water. Finally, the blocks were stored in a −80°C freezer for future use.

#### 2.2.2 Preparation of Deer Bone Meal and Nano-Deer Bone Meal

The deer bone blocks prepared above were put into a beaker and soaked in ether for 24 h to remove grease; after the ether was poured out, the blocks were soaked in absolute ethanol for 24 h for dehydration and then freeze-dried in a vacuum freeze dryer. First, the blocks were pulverized with a pulverizer and passed through a standard sieve; BM that passed through a 20-mesh standard sieve but failed to pass through a 60-mesh standard sieve was collected, which was deer BM. The BM prepared above was ball-milled using a planetary ball mill, and the BM was placed in a 50 ml agate ball milling jar. There were two kinds of balls, large and small, and the diameter of the large ball was 0.6 cm, accounting for about 20% of the total number of grinding balls. The diameter of the small ball was 0.1 cm, accounting for about 80% of the total mass of the grinding ball. Ball milling conditions were as follows: the ratio of ball to material was 4:1, the ball milling time was 12 h, and the ball milling speed was 550 r/min. The deer BM collected after ball milling was nBM.

#### 2.2.3 Preparation of Calcined Deer Bone Meal and Nano-Calcined Deer Bone Meal

The BM prepared above was spread in a ceramic bowl, calcined in a muffle furnace, slowly heated to 800°C at a rate of 1.5°C/min, maintained for 6 h, and then cooled to room temperature naturally. Then, it was passed through a standard sieve, and BM that passed through a 20-mesh standard sieve but not a 60-mesh standard sieve was collected, which was calcined deer BM (CBM). The CBM prepared above was ball-milled using a planetary ball mill. The conditions were the same as those for the preparation of nBM. The deer bone powder collected after ball milling was nCBM.

#### 2.2.4 Preparation of Demineralized Bone Matrix and Nano-Demineralized Bone Matrix

DBM was prepared by the modified Urist method (Grgurevic et al., 2017). The prepared deer bone pieces were decalcified with a concentration of 0.6 mmol/L hydrochloric acid; the mixture was stirred and soaked for 72 h, and the hydrochloric acid was replaced every 12 h during the soaking process. It was ensured that the bone pieces were fully in contact with the hydrochloric acid. The bone pieces were rinsed with sterile distilled water 3–5 times and soaked overnight to fully remove the hydrochloric acid. Then, it was soaked in absolute ethanol for 2 h for dehydration, and the ethanol was removed and ether added for 2 h. Next, the ether was removed, and the bone pieces were volatilized in a fume hood overnight; the pieces were then freeze-dried in a vacuum freeze dryer. Preliminary pulverization was done using a pulverizer; BM was passed through a standard sieve, and BM that passed through a 20-mesh standard sieve but failed to pass through a 60-mesh standard sieve was collected, which was DBM. The DBM prepared above was ball-milled using a planetary ball mill. Before ball milling, DBM was lyophilized with liquid nitrogen. The ball milling time was 24 h, and DBM was lyophilized with liquid nitrogen every 3 h. The ball milling conditions were the same as those for nBM. The ball-milled deer bone powder was nDBM.

### 2.3 Characterization of Nano-Deer Bone Meal

#### 2.3.1 Characterization of Nano-Deer Bone Meal

The particle size distribution of the three different bone powders was measured using a nanoparticle size analyzer (Zetasizer Nano-ZS90, China) after dispersing the powders in phosphate-buffered saline (PBS) with 100 ug/ml, and the average particle size was represented by the median particle size D50. The surface micromorphology of the different BM was examined by transmission electron microscopy (TEM, Tecnai G^2^S-Twin, United States). TEM images were taken after dispersing the powder in ethanol with 1 mg/ml. XRD measurement was performed on a Rigaku D/MAX-2250 V at Cu Kα (*λ* = 0.154056 nm) with a scanning rate of 4° min^−1^ in the 2θ range of 20–60°. Fourier transform infrared spectroscopy (FTIR, Perkin Elmer, FTIR-2000) was used to determine the chemical structure among different bone powders. For this measurement, the transmission IR spectra were recorded using KBr pellets (1 mg sample/300 mg KBr) over a range of 400–4000 cm^−1^ with 1 cm^−1^ resolution averaging over 100 scans. The component composition of different bone powders was characterized by thermal gravimetric analysis (TGA) (TA Instruments TGA500, United States). TGA was conducted at 10°C/min heating speed in an oxidative atmosphere (synthetic air, composed of 80% N_2_ and 20% O_2_). The element content of different BM was analyzed by X-ray photoelectron spectroscopy (ESCALab250i-XL).

#### 2.3.2 Basic Components of Nano-Deer Bone Meal

Mineral content: Inductively coupled plasma spectrometry, according to GB5009.268-2016. After the sample was digested, it was determined by inductively coupled plasma mass spectrometer, qualitatively determined by the specific mass of the element, and quantitatively analyzed by the external standard method.

Amino acid content: Determined by the amino acid full spectrum analyzer.

Ash content: Muffle furnace high-temperature burning method, according to GB5009.4-2016. The residual inorganic material after the sample was burned is called ash. The ash value was calculated by burning and weighing.

Protein content: Kjeldahl method, according to GB5009.5-2016. The protein in the sample was decomposed under catalytic heating conditions, and the ammonia produced combines with sulfuric acid to form ammonium sulfate. Ammonia was freed by alkalization distillation, absorbed with boric acid, and then titrated with a standard titration solution of sulfuric acid or hydrochloric acid. The nitrogen content was calculated according to the consumption of acid and then multiplied by the conversion factor, that is, the protein content.

Fat content: Soxhlet extraction method, according to GB5009.6-2016. Fats are easily soluble in organic solvents. The sample was directly extracted with a solvent such as anhydrous ether or petroleum ether, evaporated to remove the solvent, and dried to obtain the content of free fat.

Moisture content: Direct drying method, according to GB5009.3-2016. At 101.3 kPa (one atmospheric pressure) and a temperature of 101°C–105°C, the weight loss on drying in the sample was determined by the volatilization method, and the moisture content was calculated using the weight before and after drying.

### 2.4 Biosafety of Nano-Deer Bone Meal

#### 2.4.1 Systemic Acute Toxicity Test

According to the GB/T16886 standard for the systemic acute toxicity test of medical biomaterials, nBM, nCBM, and nDBM were placed in normal saline at a concentration of 0.2 g/ml, and the extracts of nBM, nCBM, and nDBM were obtained in a 37°C incubator for 12 h. Forty Balb/C mice were randomly divided into four groups, namely, nBM, nCBM, nDBM, and PBS groups, with each group having 50% males and 50% females. The mice were injected intraperitoneally at a dose of 50 ml/kg. The experimental group was injected with material extract, and the control group was injected with normal saline. After injection, the mice were raised in separate cages. The general situation, poisoning, and death of the mice were observed 1, 2, and 3 days after injection. The changes in animal body weight were monitored and hematological and biochemical indexes were detected. Toxicity was evaluated according to the following indicators of animal reaction degree: 1) Nontoxic: the animals were in good general condition and had no obvious signs of poisoning. 2) Mild poisoning: The activities of the experimental animals were normal, but mild dyspnea and abdominal irritation occurred. 3) Obvious poisoning: The experimental animals showed symptoms of difficulty in breathing, severe abdominal irritation, decreased activity and food intake, and mild weight loss. 4) Severe poisoning: cyanosis, tremor, respiratory failure, etc., appeared in experimental animals, and their body weight decreased significantly. 5) Death: The experimental animals died after injection.

#### 2.4.2 Skin Sensitization Test

According to the GB/T16886 standard for the skin sensitization test of medical biomaterials, nBM, nCBM, and nDBM were placed in PBS at a concentration of 0.2 g/ml, and the extracts of nBM, nCBM, and nDBM were obtained in a 37°C incubator for 12 h. Twenty-four guinea pigs were randomly divided into five groups—the nBM, nCBM, and nDBM groups, the negative control group, and the positive control group—with six in each group. The back of the experimental animals was shaved, and five points were injected intradermally on both sides of the scapula of the guinea pigs. The experimental group was injected with the extracts. The negative control group was injected with PBS. The positive control group was injected with 2% dinitrofluorobenzene. The local reaction of animal skin was observed 1, 2, and 3 days after injection. The degree of erythema and edema at the injection site was recorded and scored. The scoring criteria are shown in Supplementary Table S1.

#### 2.4.3 *In vitro* Hemolysis Test

Fresh blood was taken from rats and centrifuged at 2500 r/min for 6 min; the supernatant was aspirated to obtain red blood cells, which were then washed with PBS three times and diluted to a final concentration of 2% for later use. The experiment was divided into the nBM, nCBM, nDBM, PBS, and 0.8% triton groups with three test tubes in each group. A 1.5 ml centrifuge tube was taken, 1 ml material extract was first added, and then 0.5 ml diluted red blood cell suspension was added. The centrifuge tube was placed in a shaker at 37°C and 130 r/min for incubation for 1 h, and photos were taken. After centrifugation at 2000 r/min for 5 min, 150 ul of supernatant was taken and placed in a 96-well plate. The absorbance value at 545 nm (OD_545_) was measured using a multifunctional microplate tester. The hemolysis rate was calculated according to the following formula:

$$\text{Hemolysis rate }\left(\text{HR\%}\right)=\frac{ODValue\left(samlpes\right)-ODValue\left(PBS\right)}{ODValue\left(triton\;group\right)-ODValue\left(PBS\right)}\times \;100\%$$

#### 2.4.4 Cytotoxicity Assay

After ultraviolet disinfection for 4 h, nBM, nCBM, and nDBM were placed in Dulbecco’s modified Eagle’s medium (DMEM)/F12 medium at a ratio of 50 mg/ml at 120 r/min and incubated at 37°C for 24 h. After centrifugation, the supernatant was taken as the extraction solution. BMSCs were cultured on a fresh DMEM/F12 medium for 24 h; the culture medium was then sucked up, and the extract solution, gradient dilution solution (1/2, 1/4), and medium without the extract (negative control group) were added. Then, the extract was sucked up in the culture box for 24 h, and 10% CCK-8 solution was added into each well. After 2 h of incubation, the absorbance value of each well at 450 nm was measured using a multifunctional microplate analyzer to calculate the relative percentage of the cell proliferation rate:

$$\text{Cell Viability }\left(\text{\%}\right)=\frac{ODValue\left(samlpes\right)}{ODValue\left(control\;group\right)}\times \;100\%$$

### 2.5 *In Vitro* Cell Studies

#### 2.5.1 Cell Culture

Cell experiments were performed using rat BMSCs purchased from the Institute of Biochemistry and Cell Biology, Shanghai Institutes for Biological Sciences, Chinese Academy of Sciences. Cells were cultured in DMEM (Gibco) and supplemented with 10% fetal bovine serum (Gibco), 100 U ml^−1^ penicillin, and 100 U ml^−1^ streptomycin (Sigma) in a humidified incubator of 5% CO_2_ at 37°C. Before culturing, the samples were sterilized by immersing in 75% alcohol and exposing to ultraviolet light for 60 min; the samples were rinsed with sterile PBS thrice. The medium of cell culture was refreshed every other day.

#### 2.5.2 Cell Proliferation

The CCK-8 assay was employed to quantitatively evaluate the cell proliferation on different bone powders. BMSCs (2×10^4^ cells/well) were seeded in a 48-well plate and cultured for 24 h. After that, the medium was changed with different bone powders (50 ug/ml) containing medium and the cells were incubated for 24 h, then the cells were refreshed with normal medium and incubated for more 1, 3, and 7 days, respectively. At every prescribed time point, 15 μl/well CCK-8 solution was added to the well. After 2 h of incubation, 200 μl of the medium was transferred to a 96-well plate for measurement. The absorbance was determined at 450 nm using a multifunctional microplate scanner (Tecan Infinite M200). The complete cell culture medium was used as a control group, and results were averaged over three parallel samples.

Cells were stained with calcein (Calcein-AM) and propidium iodide, which fluoresce green for live cells and red for dead cells. The images were captured by a fluorescent inverted microscope (TE 2000U, Nikon).

#### 2.5.3 Cell Morphology

Cell adhesion and morphology were evaluated using phalloidin (Sigma) for live cells (red) and 4,6-diamidino-2-phenylindole (DAPI, Invitrogen) for the cell nucleus (blue). BMSCs (2×10^4^ cells/well) were seeded in a 48-well plate and cultured for 24 h. After that, the medium was changed with different bone powders (50 ug/ml) containing medium, and the cells were refreshed with normal medium and incubated for 3 days. After 3 days of culture, the cells were fixed with 4% paraformaldehyde and then stained with phalloidin and DAPI. The images were captured by a fluorescent inverted microscope (TE 2000U, Nikon).

#### 2.5.4 Cell Migration

First, 100 ul serum-free medium was absorbed into the upper chamber of the transwell chamber and hydrated for 30 min at 37°C; then, the hydration solution was discarded. Next, 200 ul serum-free medium containing BMSCs (3×10^4^ cells/well) was added into the upper chamber, and a 600 ul complete medium containing different BM was added into the lower chamber. After PBS washing, the cells in the upper chamber were fixed with 4% paraformaldehyde for 20 min. After PBS washing, cotton swabs were used to wipe the cells in the inner layer of the microporous membrane. Under dark conditions, 500 ul 0.1% crystal violet staining solution was added to each well. Staining at room temperature for 20 min, a fluorescent inverted microscope was used to observe and photograph. Images were taken from five fields randomly selected from each chamber, and cell counts were performed.

#### 2.5.5 Alkaline Phosphatase Activity Assay

ALP staining and corresponding quantitative detection were applied to assess the activity of ALP in BMSCs grown onto different BM (Wang et al., 2020; Yao et al., 2020). ALP staining was detected using the BCIP/NBT Alkaline Phosphatase Colour Development Kit (Beyotime, China). When BMSCs were cultured on different samples for 7 and 14 days, cells were washed three times with PBS, fixed with 4% paraformaldehyde for 15 min, and washed with PBS again. Then, 500 μl ALP dye solution was added into wells, and cells were dyed for at least 12 h at room temperature under dark conditions. The extra ALP dye solution was washed away with PBS, and the purple deposition was captured by a fluorescent inverted microscope (TE 2000U, Nikon). The ALP quantitative detection was tested using a modified Alkaline Phosphatase Assay Kit (Beyotime, China). After being cultured on a different BM for 7 and 14 days, BMSCs were washed with PBS three times and split by adding 200 μl of RIPA cell lysis solution, freezing at −80°C for 25 min, and thawing at 37°C. Then, p-nitrophenol phosphate substrate and BCA solution (Beyotime, China) were added, followed by incubation in the dark for 30 min at 37°C. The absorbances at 405 nm (OD405) and 562 nm (OD562) were read using a multifunctional microplate scanner (Tecan Infinite M200). The corresponding ALP quantitative evaluation was calculated according to the eq. OD 405/OD 562.

#### 2.5.6 Alizarin Red Staining for Calcium Deposition

Alizarin red staining and calcium deposit quantification were applied to evaluate mineral deposition in BMSCs cultured on different BM based on a previously published protocol (Li et al., 2019b; Mahmoud et al., 2020). When cells were incubated with different BM for 14 and 21 days, the cells were washed three times with PBS, fixed with 4% PFA for 15 min, and washed with PBS again. Afterward, the cells were immersed in 1% (w/v) Alizarin Red S (ARS, Sigma) solution for 30 min at 37°C. The excess ARS dye was flushed out with PBS, and the presence of mineral deposition was qualitatively evaluated according to the red color intensity observed using an inverted microscope (TE 2000U, Nikon). Furthermore, calcium quantification was performed using a 10% cetylpyridinium chloride (CPC) solution. ARS-stained cells were rinsed with PBS and then treated with 1 ml CPC solution for 1 h to desorb calcium ions. Absorbance was measured at 540 nm using a multifunction microplate scanner (Tecan Infinite M200).

#### 2.5.7 Gene Expression Analysis *via* Quantitative Reverse Transcription-Polymerase Chain Reaction

Quantitative reverse transcription-polymerase chain reaction (qRT-PCR) was introduced to further investigate the expression levels of runt-related transcription factor 2 (RUNX2), collagen type 1 (COL-1), osteopontin (OPN), and bone morphogenetic protein 2 (BMP-2). TRIzol (Takara Biomedical Technology Co., Ltd., Beijing, China) was used to extract the total RNA of the cells after 7 and 14 days of culture, and reverse transcription was performed according to the PrimeScript™ RT reagent kit with gDNA Eraser (Takara Biomedical Technology Co., Ltd., Beijing, China) to generate the template; it was then mixed with TB Green^®^ Premix Ex Taq™ (Takara Biomedical Technology Co., Ltd., Beijing, China) for PCR on a LightCycler^®^480 instrument (Roche Diagnostics Ltd., Shanghai, China). The target gene primer sequences are shown in Supplementary Table S2. The resulting mRNA levels were normalized to GAPDH and compared with those of the control group using the 2^−ΔΔct^method.

#### 2.5.8 Western Blotting Assay

BMSCs were seeded onto different BM in 6-well culture plates at a density of 15×10^4^ cells per 2 ml medium per well and then incubated for 7 days. Cells were scraped from different samples and lysed with RIPA buffer (Biosharp, China) containing phenylmethylsulfonyl fluoride. Lysates were freeze/thawed for three cycles and centrifuged at 12,000 rpm for 20 min at 4°C, and the supernatant was collected. The total protein concentration was determined using the BCA Protein Assay Kit (Thermo, United States) afterward. The loading buffer was added to the supernatant, and the mixture was heated to 100°C for 5 min. The protein sample lysates were loaded on a 12% sodium dodecyl sulfate-polyacrylamide gel and transferred to polyvinylidene difluoride membranes (Solarbio, China). The membrane was blocked with 5% skim milk in TBST buffer (0.1 M Tris-HCl and 0.1% Tween-20, pH 7.4) for 1 h and incubated with primary antibodies overnight at 4°C. Finally, the membrane was incubated with secondary antibodies at room temperature for 1 h. Protein bands were detected using the ECL Western Blotting Substrate (KF005, Affinity) and revealed using a chemiluminescence imaging system (Amersham Imager 600, GE, United States). Band densitometry was analyzed using ImageJ software. β-actin was used as the internal control.

### 2.6 Statistical Analysis

The data presented are the mean (standard deviation ± STD). Independent and replicated experiments were used to analyze the statistical variability of the data via one-way analysis of variance, with *p* < 0.05 being statistically significant (*p < 0.05).

## 3 Results and Discussion

### 3.1 Preparation and Characterization of Nano-Deer Bone Meal

#### 3.1.1 Characterization of Nano-Deer Bone Meal

**FIGURE 1:**
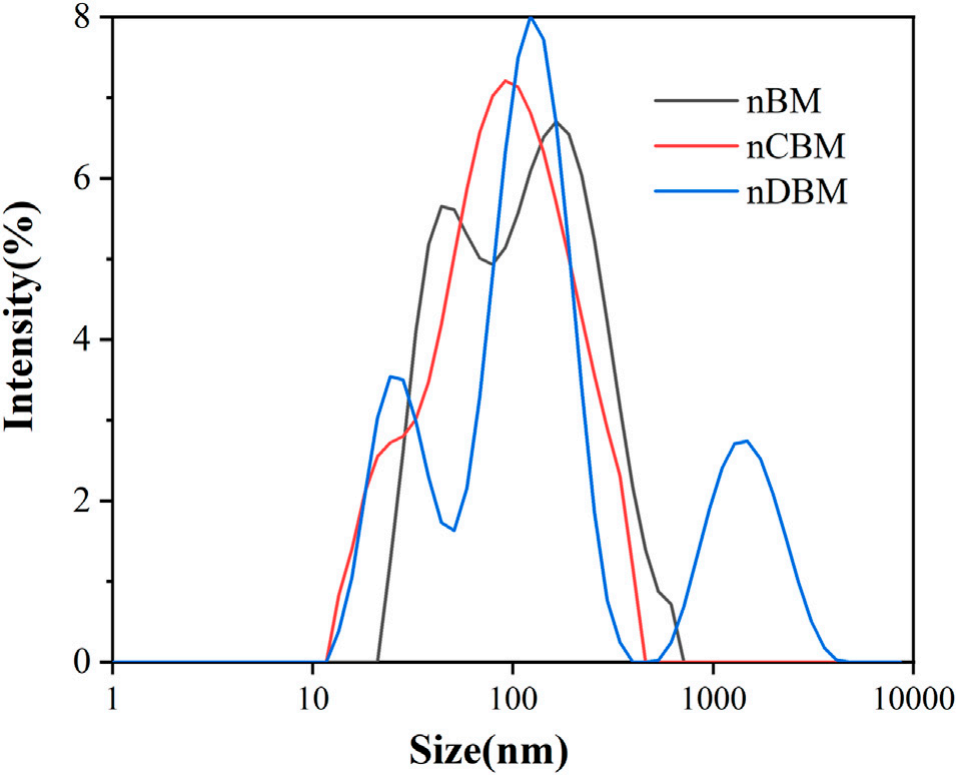
Particle size distribution of different nano bone powders.

The BM, CBM, and DBM prepared in this study all passed the screening of 20-mesh and 60-mesh standard sieves; therefore, the particle sizes of the final prepared BM, CBM, and DBM were all in the range of 250 um–850 um. In this study, nanoscale bone powder was prepared by physical ball milling of BM, CBM, and DBM. The average particle size of the three different nano bone powders is shown in Figure 1—D50 (nBM) = 106 nm (PDI = 0.372), D50 (nCBM) = 91.3 nm (PDI = 0.231), and D50 (nDBM) = 122 nm (PDI = 0.426). Nanoparticles are prepared via physical and chemical preparation methods. Physical ball milling is widely used because of its advantages of simple operation and large production (Yin et al., 2015). The particle size of prepared nanoparticles is affected by many factors, such as the ratio of ball to material, grinding time, and grinding speed. Nano-sized BM was successfully prepared under the conditions of ball milling in this study.

**FIGURE 2:**
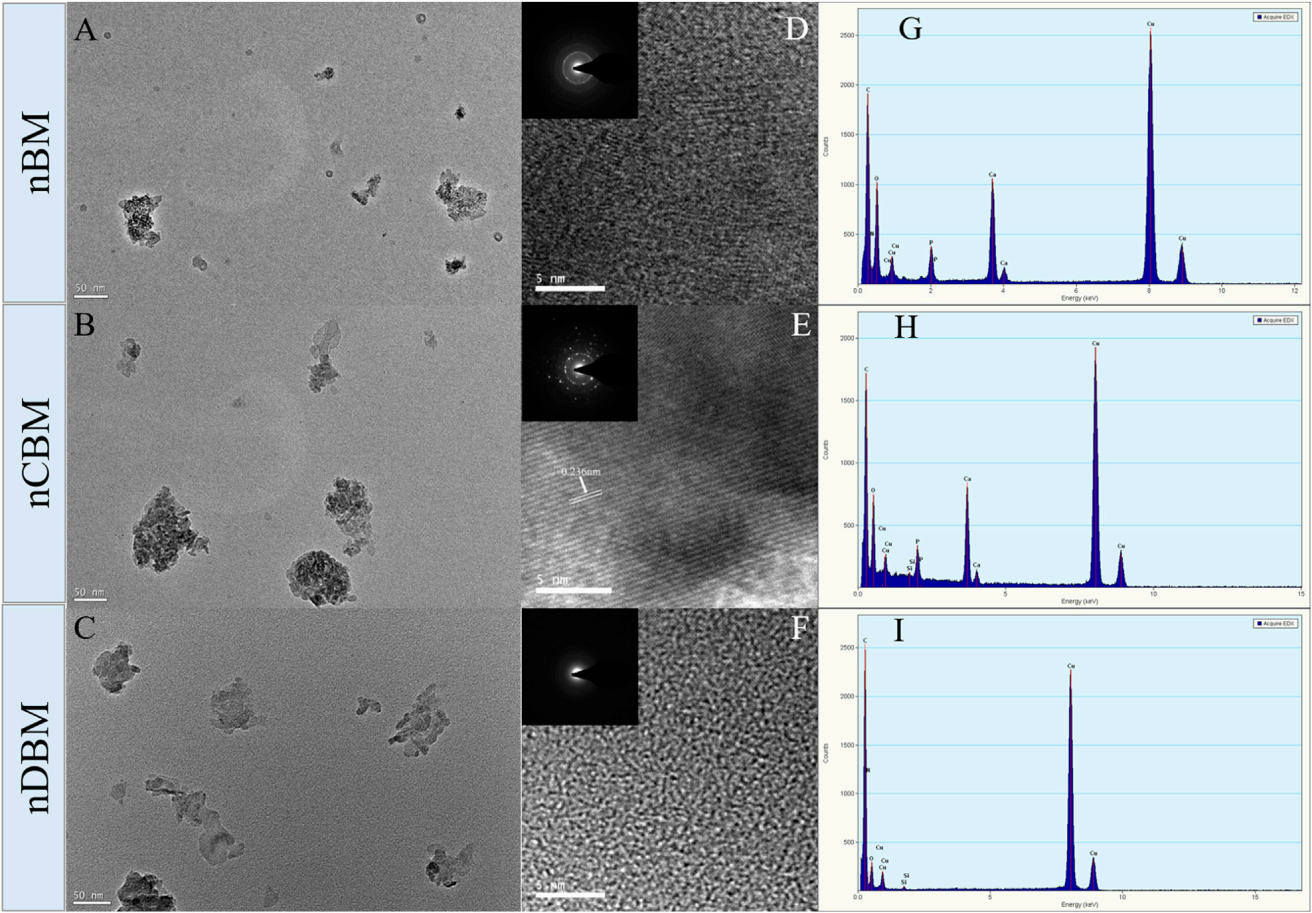
Transmission electron microscopy (TEM) image **(A,B,C)**, high-resolution TEM image **(D,E,F)**, and energy-dispersive X-ray spectroscopy spectrum **(G,H,I)** of different nano bone powders. Insets are the corresponding selected area electron diffraction patterns.

Microscopically, natural bone tissue is composed of an organic collagen fiber structure and an inorganic apatite framework structure. The basic units of the inorganic structure are needle and columnar apatite crystals. They are intertwined with amorphous collagen fibers, oriented or coiled to form a variety of structures and different functional units (Kumar et al., 2016; Wubneh et al., 2018). As shown in Figures 2A–C, nBM had an irregular shape, consisting of needle-stick hydroxyapatite crystals and an amorphous fiber structure, which completely retains the natural bone tissue structure. nCBM was calcined at a high temperature during the preparation process, and the organic collagen fibers in the bone tissue were lost; thus, the morphology was a needle-shaped hydroxyapatite. nDBM had an amorphous fibrous structure because most of the inorganic calcium and phosphorus salts were removed during the preparation process. The corresponding HRTEM images are shown in Figures 2D–F. The interplanar distances measured in the segments (fringes) of the HRTEM micrograph of nCBM were ∼0.236 nm, corresponding to the interplanar spacing of the (212) plane of the hydroxyapatite. This reveals that nCBM had a compact crystal structure. The HRTEM micrographs of nBM and nDBM suggested that they had no obvious crystal structure. These differences might be attributed to the presence of organic matrix in nBM and nDBM. The corresponding selected area electron diffraction patterns demonstrated similar results. Meanwhile, all the major elements (Ca, P, and N) are shown in the energy-dispersive X-ray spectroscopy spectrum (Figures 2G–I). nCBM did not contain N due to the lack of organic components. Because of the removal of inorganic calcium and phosphate salts, nDBM did not have Ca and P. Since nBM had both inorganic and organic components, Ca, P, and N elements appeared in the energy spectrum.

**FIGURE 3:**
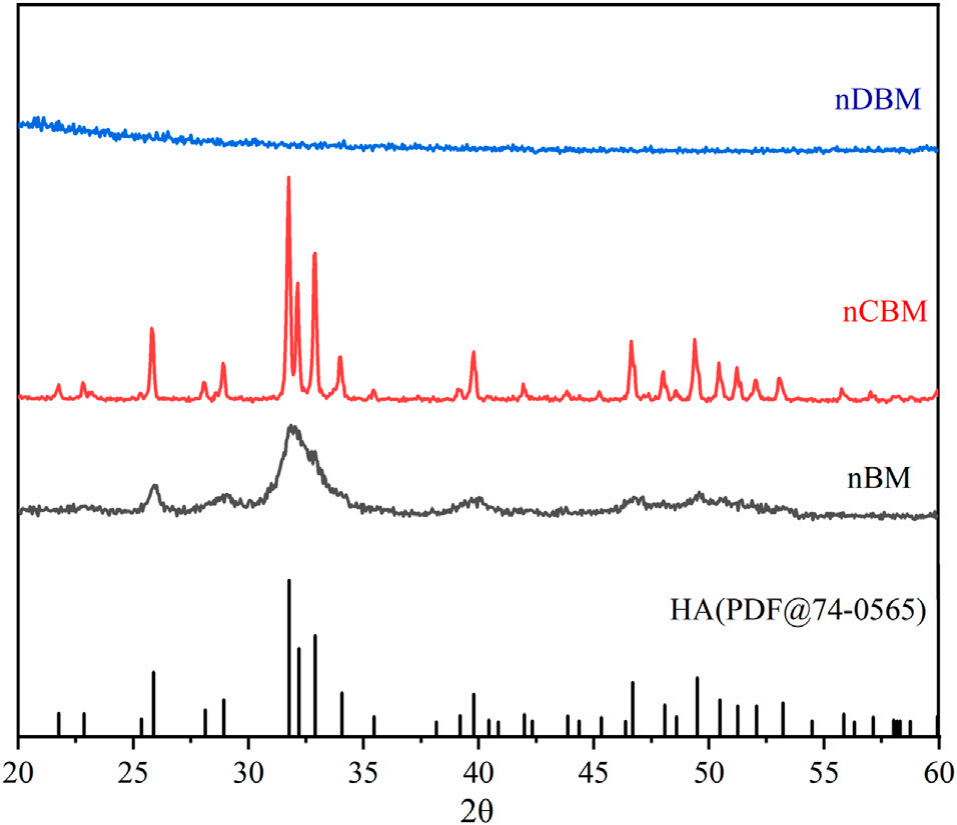
XRD patterns of different nano bone powders.

Figure 3 shows the XRD patterns of different nano bone powders. Characteristic peaks of nCBM corresponded to the major diffraction peaks of stoichiometric hydroxyapatite (PDF@74–0565). nBM presented a lower intensity and a higher signal-to-noise ratio than nCBM, while nDBM presented an amorphous band. These differences might be attributed to the presence of organic matrix in nBM and nDBM. The XRD results were in good agreement with the TEM observations.

**FIGURE 4:**
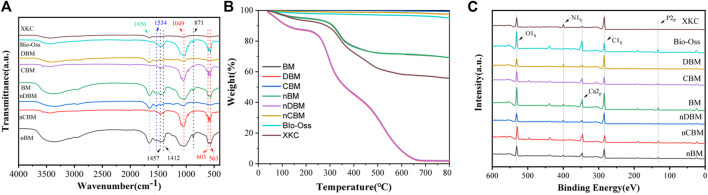
Fourier transform infrared spectroscopy spectra **(A)**, TGA **(B)**, and XPS spectrum **(C)** of different bone powders. Red dotted line: absorption peaks of PO_4_^3−^; black dotted line: absorption peaks of CO_3_^2−^; blue dotted line: absorption peaks of amide II; green dotted line: absorption peaks of amide I.

To study the differences in the chemical structures of different BM and the same kind of BM with different particle sizes, FTIR analysis was conducted. As shown in Figure 4A, 563 and 603 cm^−1^ were the bending vibration absorption peaks of PO_4_^3−^ in hydroxyapatite, 871 cm^−1^ was the bending vibration absorption peak of CO_3_^2−^, 1049 cm^−1^ was the symmetrical stretching absorption peak of PO_4_^3−^, and 1412 and 1457 cm^−1^ were the stretching vibration absorption peaks of CO_3_^2−^ (Khoo et al., 2015; Yin et al., 2015). Since most of the inorganic calcium and phosphorus salts were removed in DBM and nDBM, the two BM did not have the above characteristic absorption peaks. For the rest of the bone powders, the above characteristic absorption peaks were detected, and the peak positions of different bone powders were consistent, indicating that no new substances appeared during the ball milling process; in addition, the differences in particle size did not cause differences in the chemical structure of bone powder. Of note, 1534 cm^−1^ was the N–H bending vibration absorption peak of amide II, and 1656 cm^−1^ was the C=O stretching vibration absorption peak of amide I (Boutinguiza et al., 2012). Since CBM, nCBM, and Bio-Oss did not contain organic collagen fibers, there were no two characteristic absorption peaks. It could be seen that the chemical structures of CBM, nCBM, and Bio-Oss were mainly phosphate and carbonate, and the chemical structures of DBM, nDBM, and XKC were mainly organic. BM and nBM had both inorganic and organic chemical structures.

The proportion of inorganic and organic compounds in different bone powders could be determined by TGA. As shown in Figure 4B, after a high temperature of 800°C, the remaining substance of BM was inorganic. Therefore, CBM, nCBM, and Bio-Oss were almost all inorganic, accounting for 99.29%, 97.50%, and 94.93%, respectively. In DBM and nDBM, the proportion of inorganic matter was 1.72% and 2.08%, respectively. The proportion of inorganic matter in BM, nBM, and XKC was 69.24%, 69.23%, and 55.74%, respectively.

The elemental composition and structure of different BM were further analyzed by XPS. As shown in Figure 4C, CBM, nCBM, and Bio-Oss hardly contained organic collagen fibers, so there was no characteristic absorption peak of N element in the XPS energy spectrum. DBM and nDBM hardly contained inorganic calcium and phosphorus salts, so the characteristic absorption peaks of Ca and P elements in the spectrum were very small. Since BM, nBM, and XKC had both inorganic and organic components, characteristic absorption peaks of Ca, P, and N elements appeared in the XPS energy spectrum.

#### 3.1.2 Basic Components of Nano-Deer Bone Meal

As shown in Table 1, different particle sizes of the same type of BM did not have significant differences in the content of the basic components of BM (*p* > 0.05). This is consistent with the results of the FTIR analysis and TGA. Due to the different preparation processes, the content of the basic ingredients in different bone powders varied greatly. As is widely known, natural bone tissue is mainly composed of organic components (35%) and inorganic components (65%). We found that the proportion of ash in BM and nBM prepared in this study was higher than that in natural bone tissue and the commercial product XKC, while the proportion of protein and fat was lower than that in natural bone tissue and XKC. The ash ratio of CBM and nCBM was also higher than that of Bio-Oss. The different contents of these basic components may have different effects on the proliferation, differentiation, and migration of BMSCs.

**TABLE 1:** Basic component content of different bone powders (%).

	Ash	Protein	Fat	Moisture
BM	68.83±4.14	23.88±1.94	1.69±0.21	5.95±0.88
nBM	69.99±3.54	22.19±1.84	1.64±0.03	5.92±0.2
CBM	99.29±0.38	0	0	0
nCBM	97.50±1.22	0	0	0
DBM	2.34±0.29	81.28±3.19	2.19±0.15	13.82±0.49
nDBM	2.08±0.09	80.5±4.92	2.45±0.11	14.5±0.68
Bio-Oss	94.93±4.33	0	0	0
XKC	55.74±2.96	36.29±1.8	3.21±0.22	4.69±0.29

**FIGURE 5:**
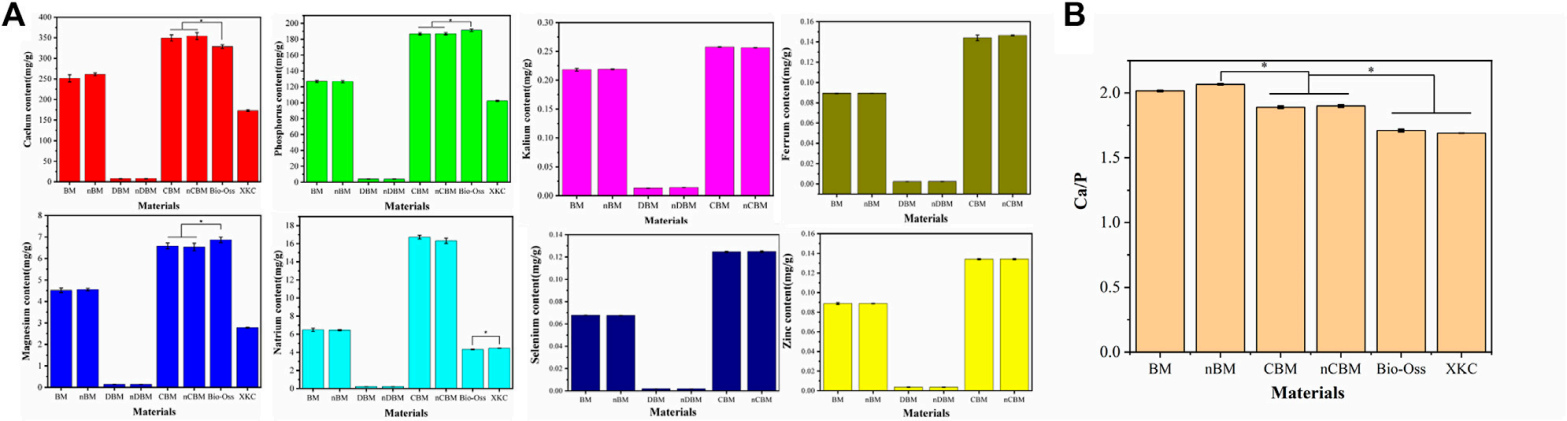
Mineral content **(A)** and Ca/P ratio **(B)** of different bone powders, *p < 0.05.

Subsequently, we measured and analyzed the main mineral elements of different BM, including calcium, phosphorus, magnesium, sodium, potassium, iron, selenium, and zinc. As shown in Figure 5A, different particle sizes of the same type of BM did not have significant differences in the mineral content of BM (*p* > 0.05). The mineral content of different BM varied greatly. In particular, the calcium content in CBM and nCBM was significantly higher than that in Bio-Oss, while the phosphorus and magnesium content was significantly lower than that in Bio-Oss (*p* < 0.05). Potassium, iron, selenium, and zinc were not detected in Bio-Oss and XKC. Therefore, by measuring the mineral content, we found a clear difference in the composition of deer bone and cow bone. In addition, we calculated the Ca/P ratio. As shown in Figure 5B, the Ca/P ratio in CBM and nCBM was significantly higher than that in Bio-Oss, and the Ca/P ratio in BM and nBM was significantly higher than that in CBM and nCBM, both of which were much higher than the Ca/P ratio in human bone tissue. Therefore, the Ca/P ratio in deer bone is significantly higher than that in cow bone and human bone tissue. Deer bone contains trace elements beneficial to the human body, which cow bone does not have; this may be the reason why deer bone can be and has been used as a traditional Chinese medicine for osteogenesis from ancient times to the present.

We selected nBM, nDBM, and XKC to determine the amino acid content. As shown in Table 2, different BM have different amino acid contents due to different preparation processes. Seven amino acids are essential amino acids (with *) and are necessary for human development. An increasing number of studies has shown that amino acids play an important role in the function of osteoblasts. It has been found that glutamine is involved in the matrix calcification process of calvarial precursor cells (Brown et al., 2011). Glutamine can participate in the tricarboxylic acid cycle and can be converted into citric acid to promote the energy metabolism of osteogenic precursor cells (Karner et al., 2015). A recent study showed that glutamine can promote the proliferation of bone marrow stem cells and their differentiation into osteoblasts (Yu et al., 2019). Atf4, an important regulator of osteogenic differentiation, can increase the uptake of amino acids and the synthesis of collagen. In patients with Coffin–Lowry syndrome caused by Atf4 mutation, both bone mass and bone density are significantly reduced (Yang et al., 2004; Elefteriou et al., 2006). In addition, arginine can promote the osteogenic differentiation of BMSCs (Chevalley et al., 1998). BM and nBM prepared in this study have both inorganic and organic components, both of which can promote osteogenesis. Therefore, in theory, they can significantly promote the osteogenic differentiation of BMSCs.

**TABLE 2:** Amino acid composition and content of different bone powders (%).

Amino acid	nBM	nDBM	XKC
Aspartic acid	1.43±0.014	5.33±0.012	2.29±0.014
Threonine*	0.49±0.003	1.83±0.001	0.65±0.001
Serine	0.71±0.069	2.63±0.011	1.08±0.004
Glutamate	2.51±0.013	9.17±0.039	3.86±0.021
Proline	2.70±0.019	10.40±0.021	4.19±0.018
Glycine	4.93±0.005	18.86±0.048	7.96±0.032
Alanine	1.98±0.008	7.37±0.005	3.20±0.012
Valine*	0.67±0.022	2.14±0.009	1.14±0.005
Methionine	0.11±0.019	0.48±0.002	0.13±0.001
Isoleucine*	0.31±0.008	1.27±0.013	0.46±0.048
Leucine*	0.74±0.001	2.94±0.022	1.10±0.021
Tyrosine	0.20±0.005	0.84±0.007	0.20±0.003
Phenylalanine*	0.49±0.029	1.83±0.004	0.73±0.011
Histidine*	0.35±0.007	0.94±0.003	0.27±0.006
Lysine*	0.81±0.012	3.01±0.016	1.21±0.009
Arginine	1.66±0.004	6.55±0.008	2.62±0.015
Essential amino acids	3.86±0.016	13.96±0.183	5.56±0.027
Total	20.11±0.326	75.58±0.481	31.09±0.109

### 3.2 Biosafety of Nano-Deer Bone Meal

#### 3.2.1 Systemic Acute Toxicity Test

**FIGURE 6:**
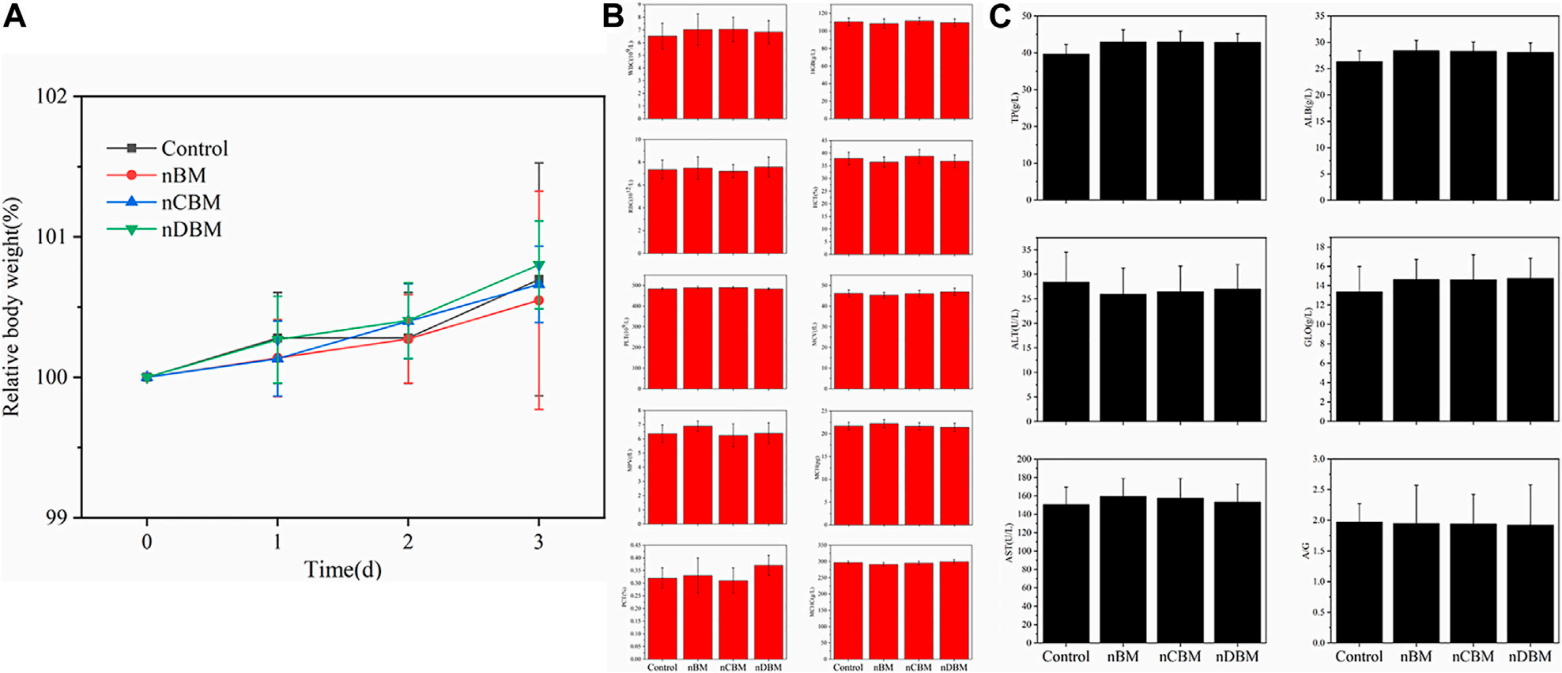
Body weight **(A)**, blood routine **(B)**, and biochemical indicators **(C)** of mice during the experiment.

The systemic acute toxicity test is performed by injecting an extract of the implant material into the blood circulation system of the animal and then observing whether the animal is poisoned, so as to judge the toxicity. General observation: At 30 min, 1, 2, and 3 days after injection, the vital signs of the mice in the experimental and control groups were good without obvious poisoning. At the same time, changes in body weight were detected. As shown in Figure 6A, the body weight of mice in the experimental and control groups increased by varying degrees, but there was no significant difference. In addition, blood routine and blood biochemical tests showed no significant difference in results after 3 days (Figures 6B and C). Therefore, the systemic acute toxicity experiments showed that the deer BM prepared in this study was biologically safe.

#### 3.2.2 Skin Sensitization Test

**FIGURE 7:**
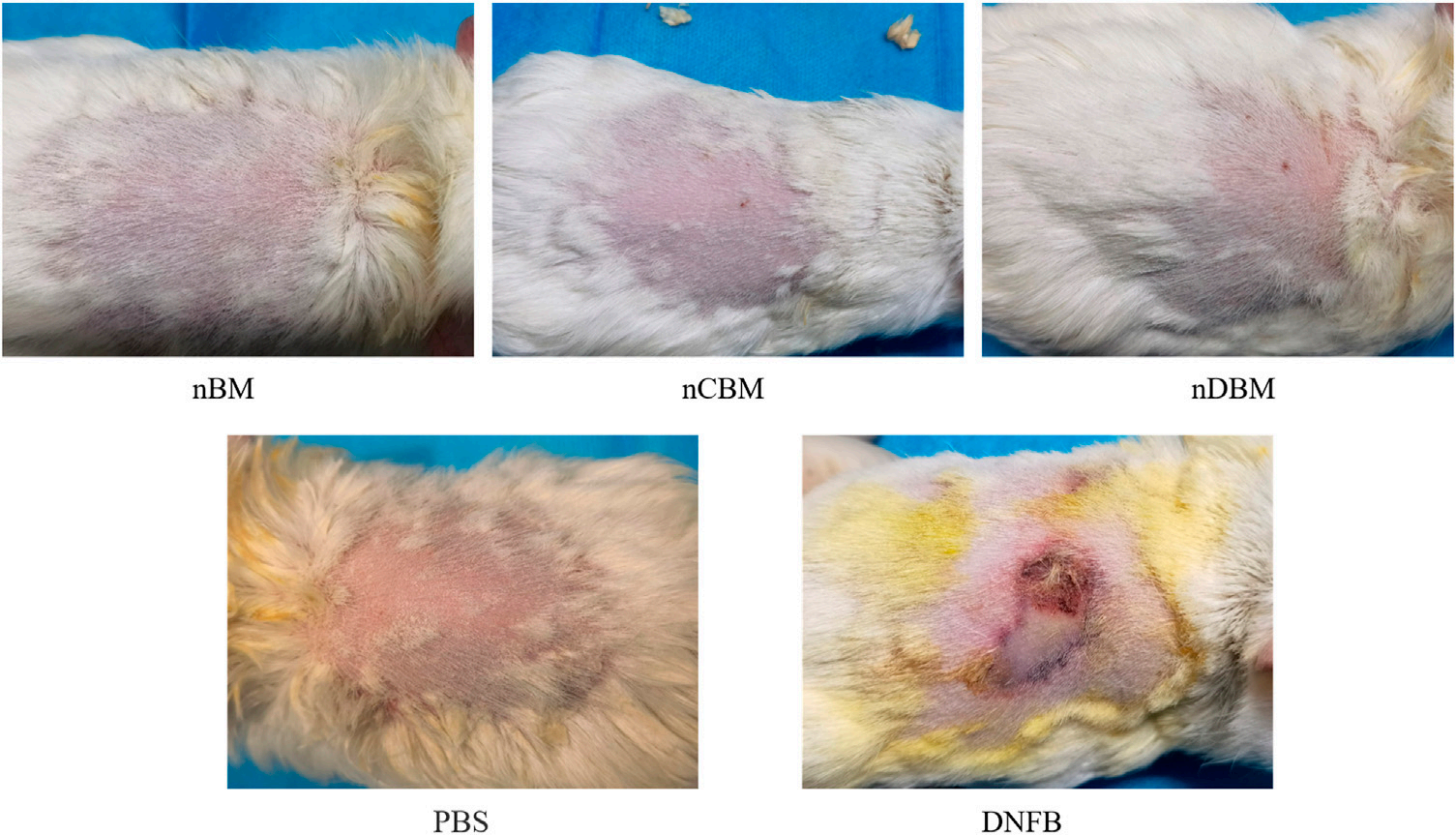
Appearance of the guinea pig skin on the 3rd day of the experiment.

The skin sensitization test is used to evaluate possible contact hazards from chemicals released from implant materials. The guinea pigs in the experimental and negative control groups did not have erythema and edema on the skin after injection of the extract, while the guinea pigs in the positive control group had severe erythema (purple) and edema (Figure 7). The scoring results are shown in Supplementary Table S3. The deer bone powder prepared in this study underwent physical and chemical decellularization treatment to remove substances that can cause immunogenic reactions. Therefore, the skin sensitization experiments showed that the deer bone powder prepared in this study does not cause contact damage to the skin.

#### 3.2.3 *In vitro* Hemolysis Test

**FIGURE 8:**
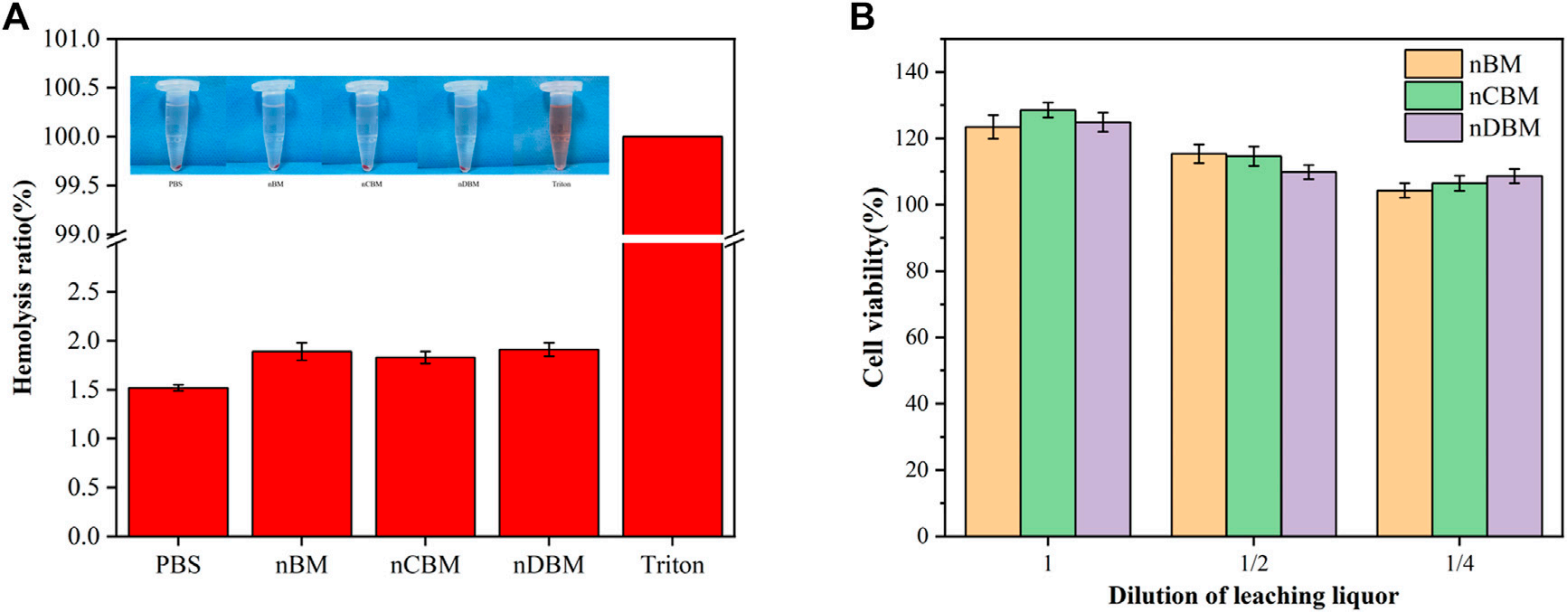
Hemolysis rate **(A)** and cytotoxicity **(B)** of different bone powders.

The *in vitro* hemolysis test is used to evaluate the effect of implanted material on the blood by detecting the rate of hemolysis after red blood cell rupture. As shown in Figure 8A, the red blood cells in the experimental and negative control groups were deposited at the bottom of the centrifuge tube, and no obvious hemolysis occurred, while the positive control group had obvious hemolysis. According to the calculation formula, the hemolysis rate of the extracts in the experimental group was less than the requirement of 5% (Figure 8A). The experimental results showed that the deer BM prepared in this study has no obvious effect on blood and meets the safety standards for medical biomaterials.

#### 3.2.4 Cytotoxicity Assay

Cytotoxicity assay is an important method to evaluate the biosafety of implant materials. As shown in Figure 8B, compared with the negative control group (medium without extract), the addition of extract and gradient diluent (1/2, 1/4) in the experimental group not only did not inhibit but also promoted the proliferation of BMSCs, and the promoting effect decreased with the serial dilution. The results showed that the deer BM prepared in this study has no cytotoxicity.

### 3.3 *In vitro* Cell Studies

#### 3.3.1 Cell Proliferation and Morphology

**FIGURE 9:**
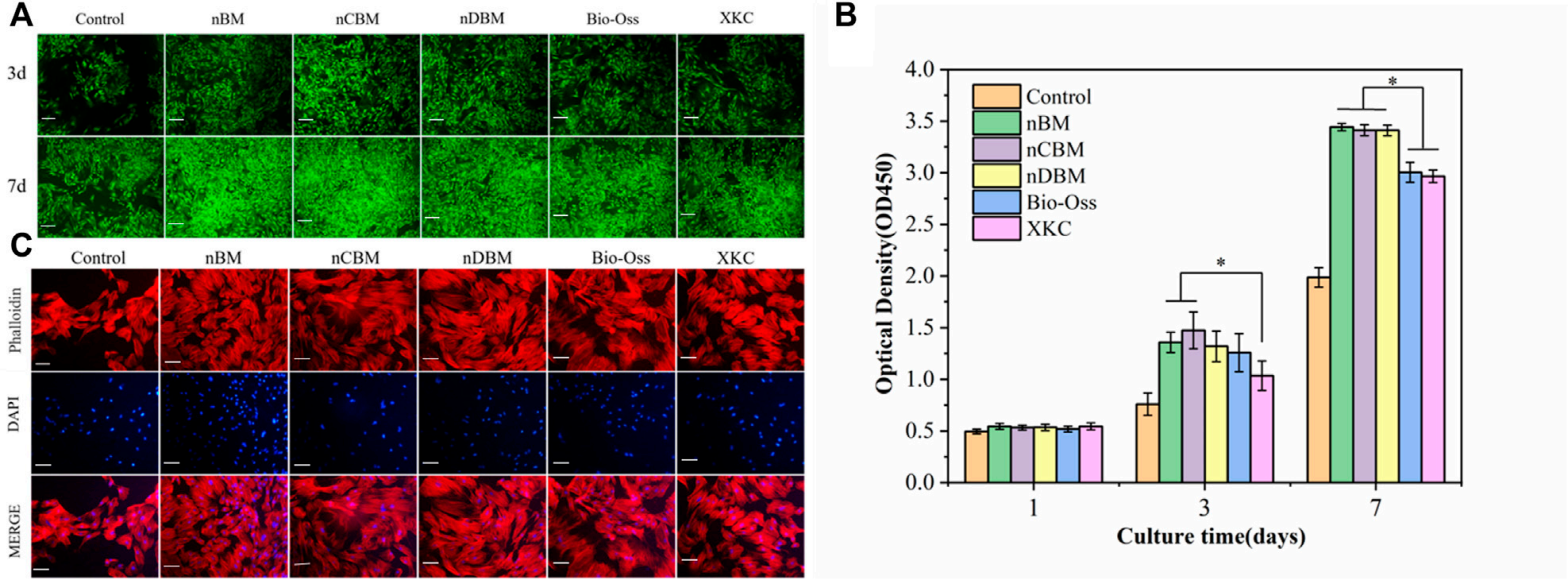
**(A)** Fluorescence of bone marrow mesenchymal stem cells (BMSCs) on different bone powders at 3 and 7 days, stained with Calcein-AM (green, live cells). Scale bars: 200 μm. **(B)** Cell proliferation of different bone powders, *p < 0.05. **(C)** Morphology of BMSCs on different bone powders at 3 days, stained with phalloidin (cytoplasm, red) and DAPI (nucleus, blue). Scale bars: 100 μm.

During bone repair, BMSC proliferation plays an important role in their function. BMSCs were cultured in media containing different BM, and the number of cells at 1, 3, and 7 days was tested using the CCK-8 method. To observe cell proliferation more intuitively, BMSCs were stained at 3 and 7 days. As shown in Figure 9B, the total number of cells in each group increased gradually with the increase in culture time. There was no significant difference in the number of cells in each group at 1 day. At 3 days, the number of cells in the BM group was significantly higher than that in the control group (*p* < 0.05). Among the different BM groups, the number of cells in the nBM and nCBM groups was significantly higher than that in the XKC group (*p* < 0.05), while the number of cells in the other BM groups was not significantly different. At 7 days, the number of cells in the nBM, nCBM, and nDBM groups was significantly higher than that in the Bio-Oss and XKC groups (*p* < 0.05), and the number of cells in all the BM groups was significantly higher than that in the control group (*p* < 0.05). As shown in Figure 9A, the cells of 3 and 7 days were stained, and almost all cells showed green fluorescence, indicating that the cells survived normally. As quantitatively analyzed, the cell density in the nBM, nCBM, and nDBM groups was significantly higher than that in the Bio-Oss and XKC groups at 7 days. In addition, when cultured for 3 days, the BMSC cytoskeleton was observed by staining, and it could be seen that BMSCs in all groups showed a typical spindle structure with a complete cytoskeleton. Compared with the sparse cells in the control group, the cells in the BM group were denser; the cells in the nBM, nCBM, and nDBM groups were more stretched and had more filamentous pseudopodia, and the connections between the cells were closer (Figure 9C). According to our analysis, on the one hand, as in the analysis of the composition of BM, the Ca/P ratio in deer bone was significantly higher than that in bovine bone and human bone tissue, and it contains trace elements beneficial to the human body, which bovine bone does not have. On the other hand, the bone powder was prepared at the nanoscale, so that the particle size becomes smaller and the specific surface area increases. In addition, nBM has a natural bone-graded structure. During the preparation process, it was subjected to strong friction and collision, which disrupts the hydroxyapatite structure deposited in the collagen fibrin; thus, it is easier to release calcium and phosphate ions. Therefore, compared with Bio-Oss and XKC, nBM, nCBM, and nDBM prepared in this study were more likely to promote the proliferation and adhesion of BMSCs.

#### 3.3.2 Cell Migration

**FIGURE 10:**
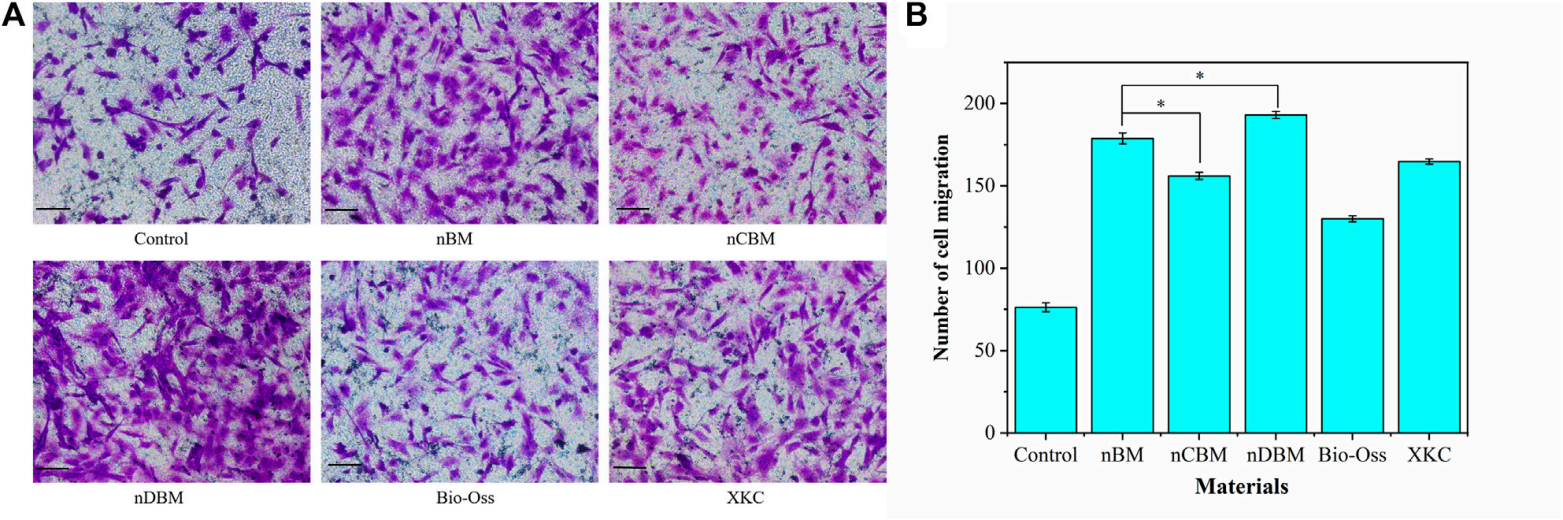
**(A)** Effects of different bone powders on the migration of BMSCs, stained with crystal violet. Scale bars: 100 μm. **(B)** Quantitative analysis of BMSC migration. *p < 0.05.

In the process of osteogenic induction, BMSCs are regulated by various factors to differentiate into osteoblasts and migrate into the area of bone regeneration. Therefore, promoting cell migration and enhancing the recruitment of BMSCs are beneficial to bone repair in bone defect areas (Chavakis et al., 2008). As shown in Figure 10A, the nDBM group had the highest cell migration density, followed by the nBM and XKC groups, and finally the nCBM and Bio-Oss groups. Quantitative analysis of the number of cells migrated showed that all BM groups significantly promoted cell migration compared with the control group (*p* < 0.05). In the BM group, the number of cells migrated in the nDBM group was significantly higher than that in the nBM and XKC groups (*p* < 0.05), followed by the nCBM group, and finally the Bio-Oss group (Figure 10B). Studies have shown that growth factors such as BMP and TGF can promote the migration of BMSCs (Gamell et al., 2011). nDBM prepared in this study contained these growth factors, which significantly promoted the migration of BMSCs. nBM and XKC were not as effective as nDBM because they contained fewer organic components. nCBM and Bio-Oss did not contain organic active ingredients, so the induction effect was not as good as that of the other three BM groups, while all were better than the control group. This is because both groups contained calcium ions, which promote cell migration. Some studies suggest that calcium ions may also be chemokines for cell migration (Liu et al., 2018). The calcium ion content in the nCBM group was higher than that in the Bio-Oss group, so the cell migration effect of the nCBM group was better than that of the Bio-Oss group.

#### 3.3.3 Alkaline Phosphatase Activity Assay

**FIGURE 11:**
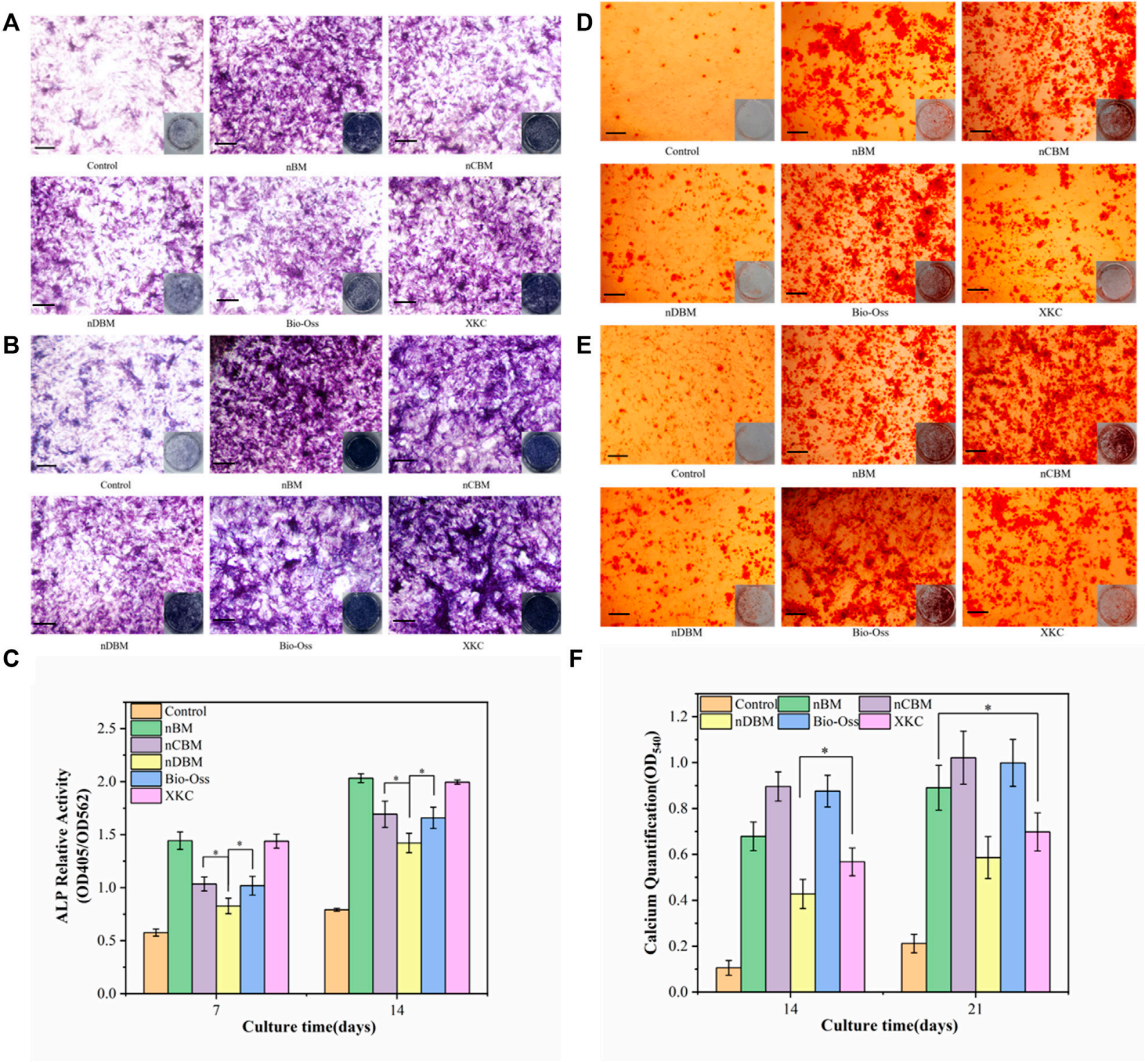
Alkaline phosphatase (ALP) staining **(A,B)** and alizarin red staining **(D,E)** in BMSCs cultured on different bone powders for 7 and 14 days (ALP) and 14 and 21 days (AR). **(C)** Corresponding quantitative evaluation of relative ALP activity in BMSCs cultured on different bone powders for 7 and 14 days. **(F)** Corresponding quantitative evaluation of calcium content and mineral deposition in BMSCs cultured on different bone powders for 14 and 21 days. Scale bars: 100 μm. *p < 0.05.

ALP is a marker of early metaphase differentiation of osteoblasts. As shown in Figures 11A and B, in general, with the increase in culture time, the ALP staining effect of each group gradually increased. When the culture time was 7 days, the staining effect of all BM groups was better than that of the control group. In the BM group, the nBM and XKC groups had the best staining results, followed by the nCBM and Bio-Oss groups, and finally the nDBM group. The trend of the staining effect at 14 days of culture was the same as that at 7 days. The ALP quantitative analysis results, presented in Figure 11C, verify the above results. After 7 and 14 days of BMSC culture, the ALP activities in the nBM and XKC groups were significantly higher than those in the nCBM and Bio-Oss groups (*p* < 0.05), and the ALP activities in the nCBM and Bio-Oss groups were significantly higher than those in the nDBM group (*p* < 0.05). Therefore, it can be concluded that nBM and XKC with both organic and inorganic components are superior to nCBM, Bio-Oss, and nDBM containing a single component in the early induction of osteogenic differentiation of BMSCs.

#### 3.3.4 Alizarin Red Staining for Calcium Deposition

Calcium deposition is a marker of osteoblast differentiation and maturation. As shown in Figures 11D and E, in general, with the increase in culture time, the color rendering effect of calcium nodule deposition in each group gradually increased. When cultured for 14 days, almost no calcium nodules were deposited in the control group, while calcium nodules were observed in all BM groups. In the BM group, the nCBM and Bio-Oss groups had the best staining results, followed by the nBM and XKC groups, and finally the nDBM group. The trend of the staining effect at 21 days of culture was the same as that at 14 days. The results of the quantitative analysis of calcium nodules, shown in Figure 11F, showed that after 21 days of BMSC culture, there was no significant difference in the number of calcium nodules among the nBM, nCBM, and Bio-Oss groups (*p* > 0.05), but the numbers in these three groups were significantly higher than those in the XKC and nDBM groups (*p* < 0.05). nCBM and Bio-Oss, which are dominated by inorganic calcium and phosphorus salts, contain a large amount of calcium nodule deposition materials, which can promote the deposition of mineralized nodules in the extracellular matrix of BMSCs. nBM also promotes the mineralization of stem cells due to its high Ca/P ratio. Therefore, nBM, nCBM, and Bio-Oss exhibited the ability to promote the osteogenic differentiation of BMSCs at a later stage.

#### 3.3.5 Gene Expression Analysis *via* Quantitative Reverse Transcription-Polymerase Chain Reaction

In the process of natural bone formation, a large number of genes related to osteogenesis are activated. These genes are considered markers in the process of osteogenesis, providing important evidence for the induction of osteogenesis by materials. The difference in cell behavior and the relative expression of osteogenesis-related genes are important evaluation indicators (Wang et al., 2004; Tang et al., 2018). To further explore the effect of different BM on the osteogenic differentiation of BMSCs at the molecular level, we used real-time quantitative PCR to quantitatively measure the osteogenic differentiation-related target genes, Runx2, Col-I, Opn, and Bmp-2. Runx2 is a key gene expressed early in the process of osteogenic differentiation, which plays an important role in the differentiation of osteoblasts, the maturation of chondrocytes, and the production of bone matrix proteins. Col-I is expressed in the early and middle stages of osteogenic differentiation of osteoblasts. It can act as a nucleation site for hydroxyapatite crystals during the mineralization stage of the extracellular matrix of osteoblasts, promote calcium deposition in the matrix, and play an important role in the mineralization of osteoblasts. Opn is a marker expressed in the middle stage of osteogenic differentiation of osteoblasts. As an important part of the extracellular matrix, it can promote the signal transduction between osteoblasts and play a key role in osteogenic induction. Bmp-2 is a growth factor that regulates the development of bone tissue; it can induce the proliferation of stem cells and their directional differentiation to osteoblasts and promote the formation of new bone (Tang et al., 2018).

**FIGURE 12:**
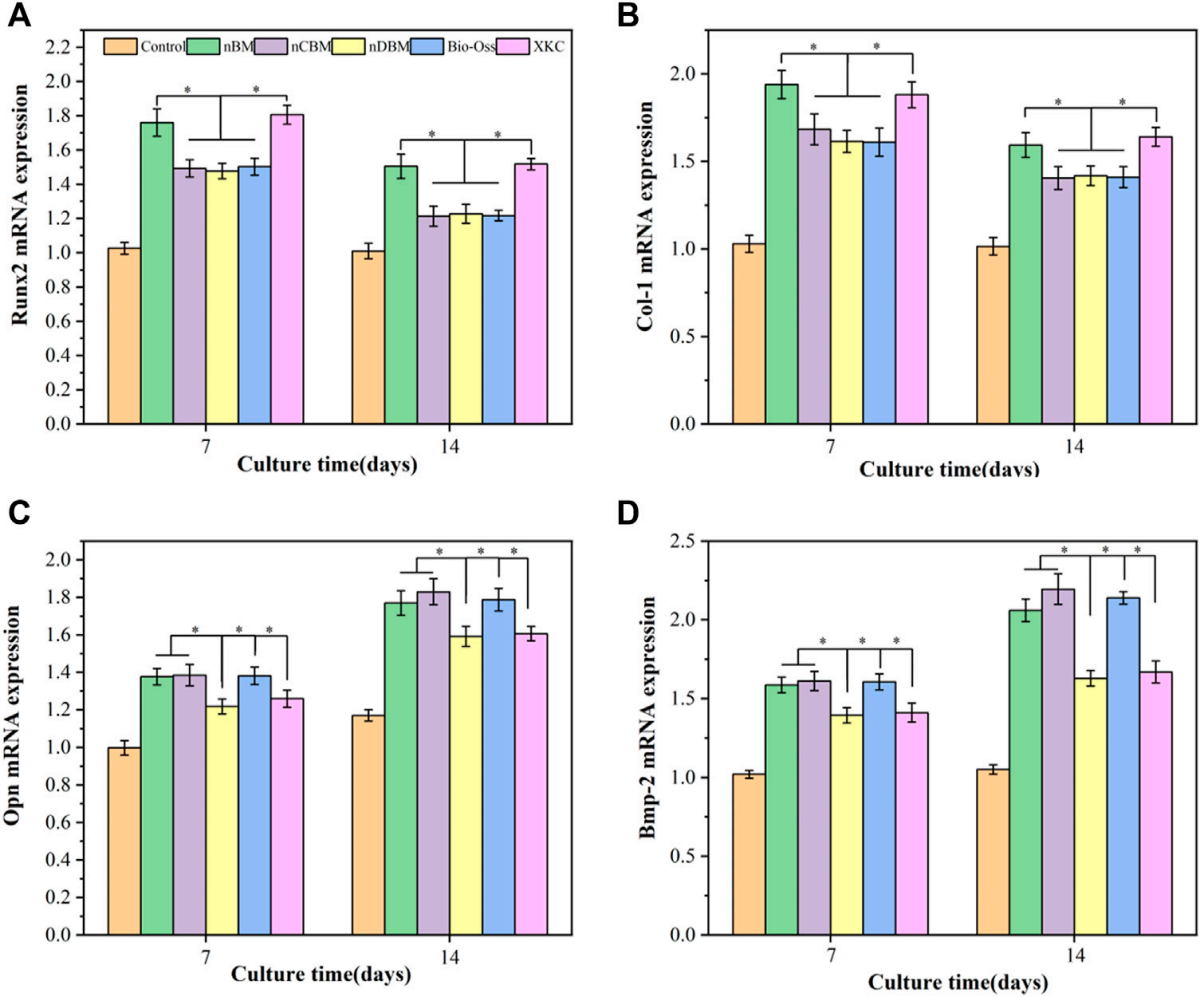
qRT-PCR analysis of Runx2 **(A)**, Col-I **(B)**, Opn **(C)**, and Bmp-2 **(D)** in BMSCs cultured on different bone powders for 7 and 14 days. *p < 0.05.

As shown in Figure 12, BMSCs were cocultured with different BM for 7 and 14 days. Compared with the blank control group, the expression levels of the Runx2 and COL-I genes in BMSCs were increased in the BM group, and the expression levels in the nBM and XKC groups were the highest, with no significant difference between the two groups. These results indicate that BM can significantly promote the early osteogenic differentiation of BMSCs, and nBM and XKC have the most significant promoting effect. Regarding Opn and Bmp-2, the expression levels of the Opn and Bmp-2 genes gradually increased with the increase in culture time. Compared with the blank control group, the expression levels of the Opn and Bmp-2 genes in the cells of the BM group increased, among which the nBM, nCBM, and Bio-Oss groups had the highest expression levels. These results indicate that BM can also significantly promote the osteogenic differentiation of BMSCs in the middle and late stages, with nBM, nCBM, and Bio-Oss showing the best effect.

#### 3.3.6 Bone-Related Protein Expression by Western Blotting

**FIGURE 13:**
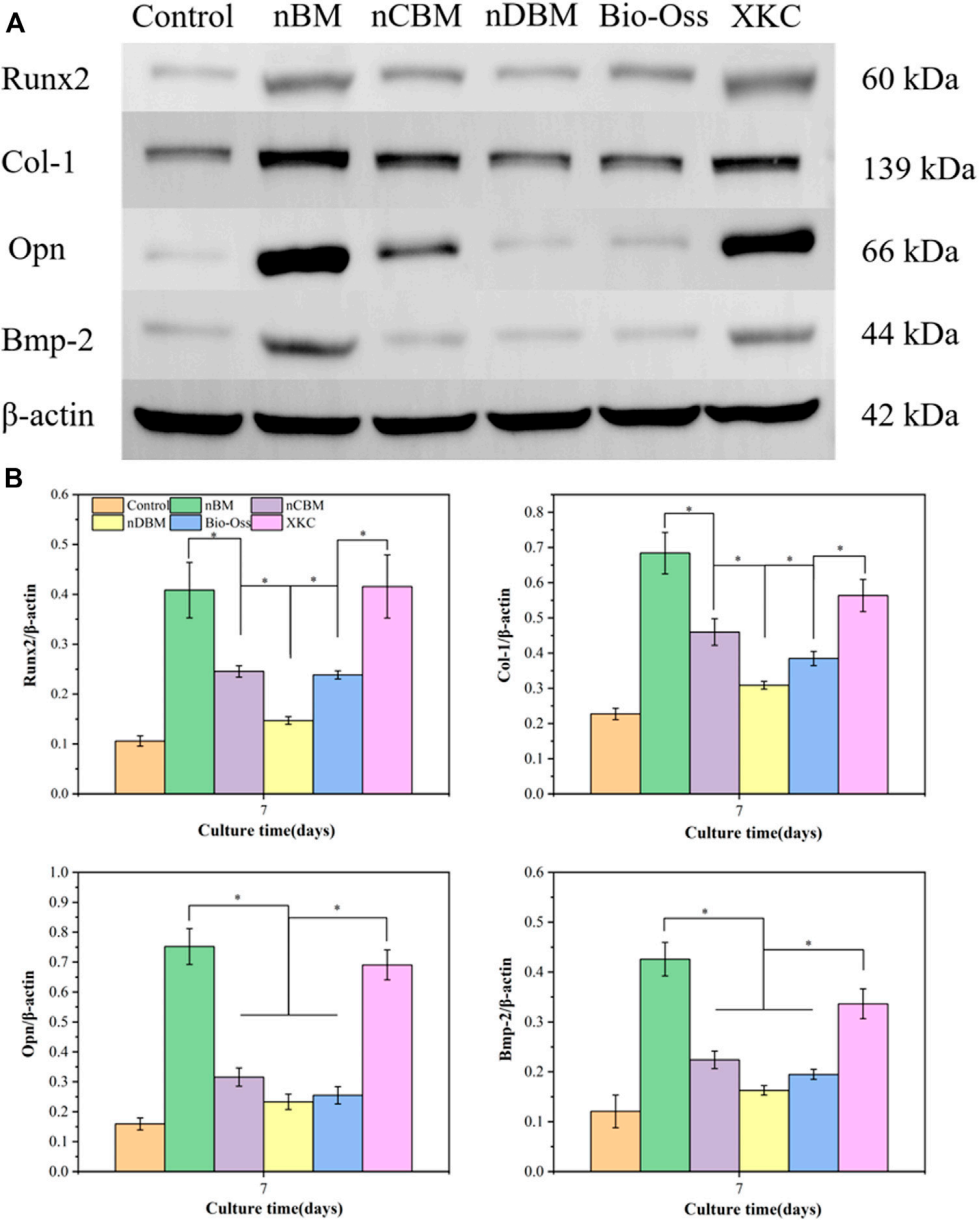
**(A)** Western blotting for protein-level detection of Runx2, Col-I, Opn, and Bmp-2 in different groups of cells for 7 days. **(B)** Band densitometry analysis of Runx2, Col-I, Opn, and Bmp-2 protein for 7 days. *p < 0.05.

To further investigate the regulation by different BM of the protein expression and osteogenic factors (Runx2, Col-I, Opn, and Bmp-2) in BMSCs, western blotting was performed, shown in Figures 13A and B. Western blotting (Figure 13A) and band densitometry (Figure 13B) analysis results indicated that compared with the blank control group, the amount of osteogenic differentiation-related proteins (Runx2, Col-I, Opn, and Bmp-2) produced by BMSCs in all BM groups increased, and the expression levels in the nBM group were the highest. The western blotting results were relatively in accordance with the observations of ALP activity, calcium mineral deposition, and expression of osteogenic-related genes. Overall, nBM can significantly promote the osteogenic differentiation of BMSCs in the early, middle, and late stages.

## 4 Conclusion

In this study, nBM, nCBM, and nDBM were successfully prepared. It was found that the Ca/P ratio in deer bone was significantly higher than that in cow bone and human bone tissue, and deer bone contained beneficial trace elements, such as potassium, iron, selenium, and zinc, which were not found in cow bone. The three kinds of deer bone powders prepared in this study had good biocompatibility and met the implantation standards of medical biomaterials. Cell function studies showed that compared with Bio-Oss and XKC, nBM, nCBM, and nDBM prepared in this study were more likely to promote the proliferation and adhesion of BMSCs. In terms of cell migration, nDBM was better than nBM and XKC, followed by nCBM, and finally Bio-Oss. nBM and XKC performed excellently in inducing osteogenic differentiation of BMSCs in the early stage, while nBM, nCBM, and Bio-Oss performed better in promoting the osteogenic differentiation of BMSCs in the later stage. Based on the above results, nBM has excellent performance in the proliferation, adhesion, migration, and differentiation of BMSCs. These findings indicate that nBM can be used as a potential osteoinductive active nanomaterial to enhance bone tissue engineering scaffolds with certain application prospects.

## Data Availability

The original contributions presented in the study are included in the article/Supplementary Material; further inquiries can be directed to the corresponding author.
