# Supplementary material for: Physical and Chemical Properties, Biosafety Evaluation, and Effects of Nano Natural Deer Bone Meal on Bone Marrow Mesenchymal Stem Cells
Source: Front Bioeng Biotechnol. 2022 Jul 15;10:891765. doi: 10.3389/fbioe.2022.891765 (PMC9335367; doi:10.3389/fbioe.2022.891765)
Supplement: Supplementary file 1 [file Table1.docx]

| Erythema and eschar formation | Score | Edema formation |
| --- | --- | --- |
| No erythema | 0 | No edema |
| Very slight erythema (barely visible) | 1 | Very slight edema (barely visible) |
| Clear erythema | 2 | Clear edema (swelling, not beyond the edge of the area) |
| Moderate erythema | 3 | Moderate edema (swelling about 1mm) |
| Severe erythema (purple red) to eschar formation | 4 | Severe edema (swelling more than 1mm and beyond the contact zone) |

**Table S1** Intradermal reaction scoring criteria.

| Gene | Forward primer sequence (5-3) | Reverse primer sequence (5-3) |
| --- | --- | --- |
| Runx2  Col-I  Opn  Bmp-2  GAPDH | CCGAGACCAACCGAGTCATTTA  CCCAGCGGTGGTTATGACTT  CCAGCCAAGGACCAACTACA  AGAAAGGCAACAGAAGCCCA  CTTGTGCAGTGCCAGCCTC | AAGAGGCTGTTTGACGCCAT  TCGATCCAGTACTCTCCGCT  AGTGTTTGCTGTAATGCGCC  ACCATGGTCGACCTTTAGGAG  GATGGTGATGGGTTTCCCGT |

**Table S2** Real-time PCR primer sequences

| Materials | Score |
| --- | --- |
| nBM  nCBM  nDBM  PBS  DNFB | 0  0  0  0  8 |

**Table S3** Skin sensitization test scores of different bone powders.
